# Enhancing the quality and trustworthiness of large language model-generated summaries of clinical oncology literature

**DOI:** 10.1093/jamiaopen/ooag078

**Published:** 2026-06-16

**Authors:** Arnulf Stenzl, Eamonn Rogers, Sophia Ananiadou, Yanshan Wang, Andrew J Armstrong, Andrea Sboner, Giovanni Cacciamani, Bob J A Schijvenaars, Kausar Riaz Ahmed, Hanna Thomsen, Timothy Wiemken, Antonio Campello, Cora N Sternberg

**Affiliations:** Department of Urology, University of Tübingen, Tübingen 72076, Germany; Department of Urology, University College Hospital, Galway H91 YR71, Ireland; Department of Computer Science, The University of Manchester, Manchester M13 9PL, United Kingdom; Department of Health Information Management, University of Pittsburgh, Pittsburgh, PA 15260, United States; Departments of Medicine, Urology, Pharmacology, and Cancer Biology, Duke Cancer Institute Center for Prostate and Urologic Cancers, Duke University, Durham, NC 27710, United States; Department of Pathology and Laboratory Medicine, Weill Cornell Medicine, New York, NY 10065, United States; Institute for Computational Biomedicine, Weill Cornell Medicine, New York, NY 10021, United States; Meyer Cancer Center, Weill Cornell Medicine, New York, NY 10065, United States; Englander Institute for Precision Medicine, Weill Cornell Medicine, New York, NY 10021, United States; Keck School of Medicine, University of Southern California, Los Angeles, CA 90033, United States; Digital Science, London, EC1M 5NR, United Kingdom; Pfizer Inc., Silver Spring, MD 20910, United States; Pfizer Inc., Bothell, WA 98021, United States; Pfizer Inc., New York, NY 10001, United States; Digital Science, London, EC1M 5NR, United Kingdom; Englander Institute for Precision Medicine, Weill Cornell Medicine, New York, NY 10021, United States

**Keywords:** artificial intelligence, hallucinations, large language models, scientific summaries, oncology literature

## Abstract

**Objectives:**

This study evaluated the quality and trustworthiness of large language model (LLM)-generated scientific and plain language summaries (PLS) from clinical oncology literature, focusing on faithfulness (absence of hallucinations), relevance, and readability.

**Materials and Methods:**

Ten LLM-generated scientific summaries and PLS from the INSIDE (artificial INtelligence to Support Informed DEcision making) prostate cancer dataset. For comparison, expert-written PLS from the BioLaySumm dataset were used. A panel of 5 LLMs and 3 human experts verified faithfulness. Verification was performed on original facts and facts modified with varying levels of error (subtle, moderate, contradictory). Readability was assessed using Flesch-Kincaid Reading Ease (FRE) scores.

**Results:**

Fact verification against the summaries was ∼100%, confirming accurate fact extraction. LLM panel vs human panel agreement was substantial (kappa 0.67), outperforming agreement among the interhuman (0.43 [95% CI, 0.34–0.52]) and inter-LLM (0.40 [0.38–0.42]) panels. Large language model scientific summaries showed high faithfulness (88.9% [88.0–89.8]) and low hallucinations (9.6% [6.5–12.7]) compared to human-written PLS (61.6% [60.1–63.1] faithfulness; 40.6% [37.8– 43.4] hallucinations). The LLMs detected errors sensitively with scores decreasing as fact modifications became more severe. Finally, LLM-generated PLS were more readable than human-written versions (FRE 42.3 [interquartile range, IQR 35.27–49.41] vs 28.8 [IQR 21.02–36.18]).

**Discussion:**

A panel of LLMs reliably assessed the faithfulness of scientific summaries to their original source and thus can help increase reliability for clinical use. The lower faithfulness in human-written PLS likely reflects extrinsic hallucinations added for context.

**Conclusion:**

The study demonstrates a novel approach to automatically assess the quality and trustworthiness of LLM-generated scientific and PLS via faithfulness, relevance, and readability.

## Introduction

The rapidly growing field of natural language processing (NLP) and large language models (LLMs) have the potential to summarize complex medical literature, decreasing the burden of information overload for clinicians and scientists.^1–4^ The evolution of generative artificial intelligence (AI) has driven broad adoption of LLMs. These LLMs are now applied across a wide range of domains, including scientific research and education.^5^^,^^6^ Typically, LLMs are pretrained models using deep learning techniques through large volumes of training datasets to generate coherent human-like language outputs in response to the given instructions or prompts.^5^^,^^7^

Given the exponential growth of clinical literature, LLMs offer a powerful tool for clinicians and provide a dynamic learning experience by facilitating faster data mining and identification of relevant scientific information. This enables evidence-based decision-making and learning novel treatment algorithms for improved clinical practice.^2^^,^^8^ Therefore, the integration of LLMs into health-care systems may have several potential benefits for both health-care providers and patients.^7^^,^^9^ However, a critical limitation of current LLMs includes their tendency to generate inaccurate or fabricated data, known as hallucinations. This raises ethical concerns about the quality of their outputs, safety, and reliability.^5^^,^^7^ The quality, diversity, and size of data used to train LLMs are few of the key factors influencing the frequency of hallucinations.^7^

Hallucinations, particularly those arising in LLM-generated summaries of medical literature, pose a major challenge for impacting clinical decisions and could compromise patient safety. Critically, such hallucinations in the LLM output can affect the reliability and trust of the use of LLMs.^5^^,^^7^ Rates of hallucinations in clinical data and medical literature are not yet definitively established. Therefore, it is essential to evaluate and validate these LLMs to understand the frequency and type of hallucinations for improving the trustworthiness of clinical summaries generated by LLMs. This will facilitate the implementation of effective mitigation strategies and establish best practices or metrices accounting for such hallucinations.

This study investigated and compared the quality and trustworthiness of LLM- and human-generated summaries of clinical oncology and prostate cancer literature by identifying hallucinations. This analysis utilized faithfulness (as a representative indicator of hallucinations), relevance, and readability as metrics to assess the quality and trustworthiness of LLM-generated content. These metrics were further validated using human expert judgment as qualitative domains for characterization of hallucinations as well as for implementing best practices to improve transparency of AI-supported literature in health-care settings.

## Methods

### Data sources and LLMs

**Figure 1.**
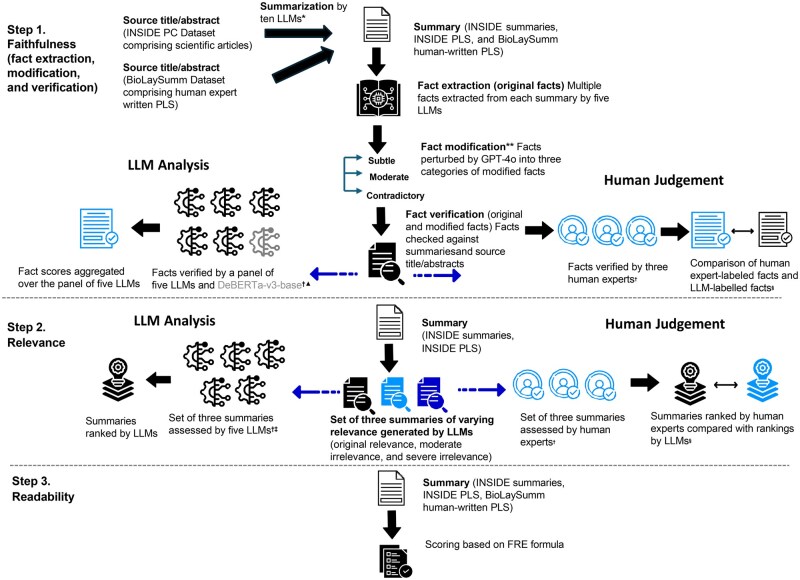
Summary of the process for assessing faithfulness, relevance, and readability. *Summarization of source title/abstracts from INSIDE dataset was performed by 10 different LLMs (GPT-4o, GPT-4o-mini, Gemini-1.5-Flash-002, Gemini-1.5-Pro-002, Claude-3-Haiku, Claude-3.5-Sonnet, Llama-3-2-1B, Llama-3-70B, Mistral-8x7B, and Mistral-Large). ^**^Fact modification by GPT-4o was only applicable to a subset of facts extracted by LLMs from the INSIDE dataset. The modified facts were verified against the summaries. ^▲^Facts verified by 5 LLMs (GPT-4o, Gemini-1.5-Pro-002, Claude-3.5-sonnet, Llama-3-70b, and Mistral-8x7B) and model based on DeBERTa-v3-base for comparison. ^†^Indicates assessment of interrater reliability between individual LLMs or human experts. ^§^Indicates inter-rater reliability among panel of LLMs and all human experts. ^‡^Five LLMs include GPT-4o, Gemini-1.5-Pro-002, Claude-3.5-sonnet, Llama-3-70b, and Mistral-8x7B. Abbreviations: FRE, Flesch-Kincaid Reading Ease; GPT, generative pretrained transformer; INSIDE, artificial INtelligence to Support Informed DEcision making; LLMs, large language models; PLS, plain language summaries.

The source title/abstracts were taken from 2 distinct datasets: INSIDE PC (artificial INtelligence to Support Informed DEcision making in Prostate Cancer) and a random sample of BioLaySumm (focused on improving lay summarization of biomedical research publications). The INSIDE PC platform serves as an alternative to PubMed and is designed to help clinicians with optimizing therapeutic sequencing for advanced PC.^4^ BioLaySumm includes plain language summaries (PLS) written by human authors on publications from PLOS and eLife datasets.^10^ While the INSIDE dataset included 108 scientific articles, the BioLaySumm dataset included 121 human-written PLS. Publications from BioLaySumm have been sampled from the broader dataset, filtered to only include documents whose title or abstract contains the words “cancer” and “patient,” in order to improve comparability between both BioLaySumm and INSIDE datasets. Publications from INSIDE use a random sample from the 3 top use cases for the INSIDE dataset.^4^ The source title/abstracts from INSIDE dataset were summarized by 10 LLMs to yield both LLM-generated scientific summaries (henceforth INSIDE summaries) and LLM-generated PLS (henceforth INSIDE PLS; Table 1). Summarization was not required for human-written PLS. A panel of 5 LLMs from different providers with distinct data training systems (GPT-4o by Open AI, Gemini-1.5-Pro-002 by Google AI, Claude-3.5-sonnet by Anthropic, Llama-3-70b by meta, Mistral-8x7B by API providers) and 5 human experts (clinical experts across multiple disciplines) measured quality and trustworthiness of the INSIDE summaries, INSIDE PLS, and human-written PLS against the source title/abstracts (Figure 1, Table 1). Besides the panel of 5 LLMs, a model based on DeBERTa-v3-base was used for fact verification (Supplementary Material S1).^5^^,^^11–13^ The interrater reliability among LLMs and human experts was assessed using Cohen’s and Fleiss kappa scores (Supplementary Material S1).^14^^,^^15^

**Table 1.** Panel of LLMs used for different steps to analyze hallucination in summaries.

Category	Models
Summarizers	GPT-4o, GPT-4o-mini, Gemini-1.5-Flash-002, Gemini-1.5-Pro-002, Claude-3-Haiku, Claude-3.5-Sonnet, Llama-3-2-1B, Llama-3-70B, Mistral-8x7B, Mistral-Large
Fact extractors	GPT-4o, Gemini-1.5-Pro-002, Claude-3.5-sonnet, Llama-3-70b, Mistral-8x7B
Fact verifiers	GPT-4o, Gemini-1.5-Pro-002, Claude-3.5-sonnet, Llama-3-70b, Mistral-8x7B, DeBERTav3-base

Specific software packages, frameworks, and their respective versions used in the experiments are listed in Supplementary Material S1 and Table S1.

### Evaluation metrices for quality and trustworthiness

This study evaluated the quality and trustworthiness of LLM-generated summaries using 3 metrics: faithfulness, relevance, and readability (Figure 1, Table S2).

#### Faithfulness: fact extraction, modification, and verification by LLMs (LLM analysis)

Faithfulness was assessed by scoring a summary if it accurately reflected the content of the source title/abstract, that is, representing statements *aka* facts extracted from the summary were compared to the source title/abstract. A statement could be factually correct but still unfaithful if it was lacking in the source. The aggregated fact scores were compared for final analysis. A series of original facts extracted from INSIDE summaries, INSIDE PLS, and human-written PLS by LLMs were checked against the corresponding summaries and source title/abstract by the 5 LLMs (Table 1) and model based on DeBERTa-v3-base to determine a hallucination as “yes” (ie, the fact is faithful and not a hallucination) or “no” (ie, not faithful indicating a hallucination) (Table 1). It is worth noting that the panel of LLMs voting was performed as an independent and decentralized process, that is, the judges did not have access to the rationale of the summary generation and did not interact with each other. This generated an aggregated fact score, ranging from 0 (all LLMs agreed the fact was a hallucination) to 100 (all LLMs agreed that the fact was faithful) which measured faithfulness (Figure 1 step 1). Hallucination rate of a document was calculated as the number of hallucinations (ie, unfaithful facts) divided by the total number of facts extracted. A fact was considered a hallucination upon agreement by 3 or more LLMs in the panel.

To evaluate if the LLMs could determine the faithfulness of modified facts and effectively assess a more diverse dataset, a subset of extracted facts from the INSIDE dataset were modified to introduce increasing levels of hallucinations (subtle, moderate, and contradictory) using GPT-4o. These facts were then verified by the 6 LLMs against the corresponding summaries to assess the faithfulness and quality of fact extraction. It was important that both the fact extraction and verification were performed accurately to ensure that the system is trustworthy (Figure 1 step 1).

#### Faithfulness: human validation (human judgment)

To further validate the fact verification workflow, up to 3 human experts (from a pool of 5 reviewers) evaluated a subset of original and modified facts for faithfulness against the summaries and source title/abstracts. This also helped to test if there was a high correlation between the LLMs and human experts. The facts were reviewed by human experts using a modified version of the annotation system, Doccano.^16^ The experts were asked to label each claim as one of the following: “Fact ok,” “Fact partly ok,” “Hallucination,” “Nonsense,” or “Unable to judge” (Table S3). Since the final assessment was based on whether a fact can be trusted or not, the individual labels were merged into 2 groups: “Fact ok” (the original “Fact ok” and “Fact partly ok”) and “Hallucination” (the original “Hallucination” and “Nonsense”). The facts labeled by human experts were then compared to those from the LLMs. The dual LLM and human evaluation ensured a rigorous assessment of the faithfulness of a trustworthy summarization system (Figure 1 step 1).

#### Relevance (LLM analysis and human judgment)

To assess relevance, the panel of 5 LLMs and 3 human experts (from a pool of 5 reviewers; each summary was presented to 3 experts) scored INSIDE summaries against the source title/abstracts. For this analysis, only LLM-generated summaries (3 scientific and 3 PLS for each summary) from 50 INSIDE abstracts were used. Since the goal of this study was to evaluate LLM-generated summaries, human-written PLS were excluded from the assessment for relevance. To create a challenging dataset, each of the summaries were modified to generate a set of 3 versions of varying relevance (A, B, and C), 2 of which lacked important information or had irrelevant details, reducing their relevance compared to the third version (original summary). For this, the prompt modifications used were original (as relevant as possible), moderate irrelevance, and severe irrelevance (Table S4). Each set of 3 summaries and the source title/abstract was presented to the 5 LLMs and human experts to rank (“triplet ranking”; Figure 1 step 2). The goal was to assess if the LLM panel would generate a similar judgment as the human experts. The rankings from human reviewers were used to construct a ranking table.

#### Readability

Readability was measured using the Flesch-Kincaid Reading Ease (FRE) formula (Supplementary Material S1).^17^^,^^18^ No panel of LLMs or human experts were used for this assessment (Figure 1 step 3).

### Summary display

To increase transparency and trust among readers regarding hallucinations, which in principle are unavoidable, we propose to present the output for summaries annotated in 2 ways: (1) a visualization showing number of scoring elements regarding the various metrics analyzed (eg, 90% for faithfulness, 45% on readability, and 80% on relevance) and (2) annotations in the summary indicating facts if not faithful to the original source.

## Results

### Faithfulness: fact verification by LLMs (LLM analysis)

**Figure 2.**
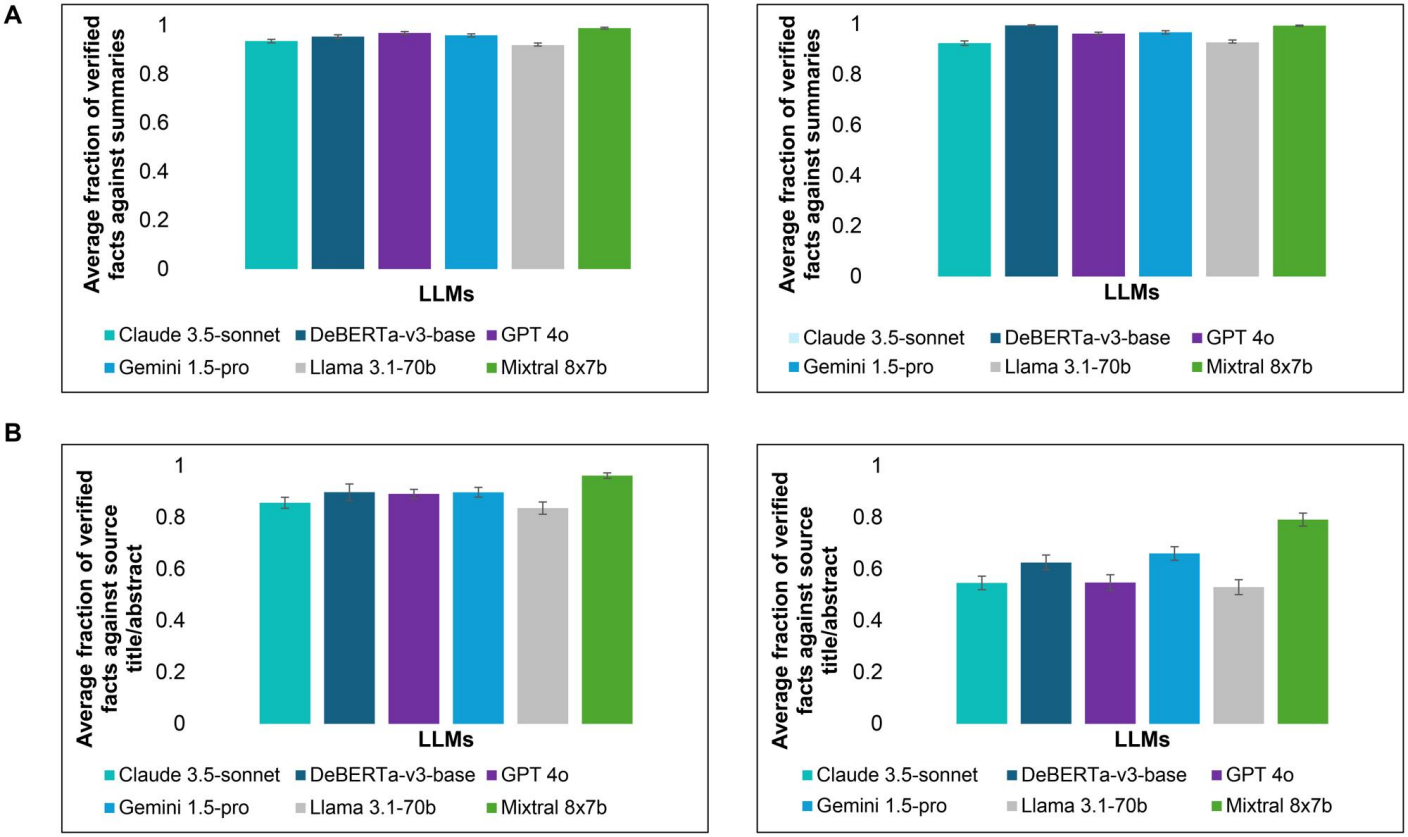
Fact verification using LLMs on INSIDE and BioLaySumm datasets. Facts verified by 5 LLMs (GPT-4o, Gemini-1.5-Pro-002, Claude-3.5-sonnet, Llama-3-70b, and Mistral-8x7B) and model based on DeBERTa-v3-base for comparison. (A) Fact verification scores from the LLM-generated summaries and PLS from INSIDE and BioLaySumm dataset, respectively. This assessment was done to check if the fact extraction step was working as expected. (B) Actual fact verification from original source title/abstract from INSIDE and BioLaySumm dataset, respectively. Abbreviations: INSIDE, artificial INtelligence to Support Informed DEcision making; LLMs, large language models.

**Figure 3.**
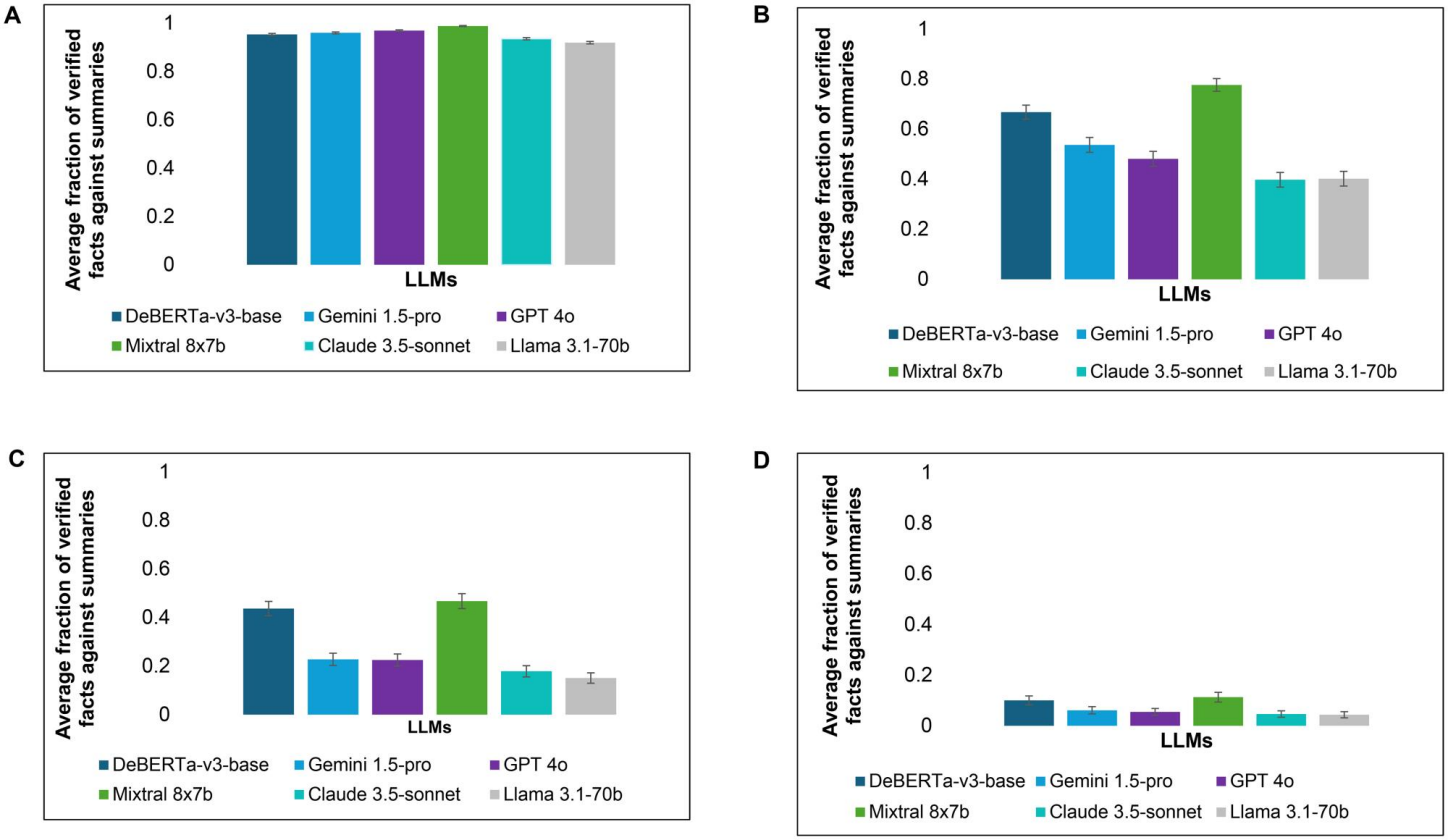
Fact verification using LLMs on perturbed facts against summaries. Facts verified by 5 LLMs (GPT-4o, Gemini-1.5-Pro-002, Claude-3.5-sonnet, Llama-3-70b, and Mistral-8x7B) and model based on DeBERTa-v3-base for comparison. (A) Fact verification scores for original facts against the LLM-generated summaries. (B) Fact verification scores for the subtle modification facts against the LLM-generated summaries. (C) Fact verification scores for the medium modification facts against the LLM-generated summaries. (D) Fact verification scores for the contradictory facts against the LLM-generated summaries. Abbreviation: LLMs, large language models.

A total of 226 summaries (INSIDE 105; BioLaySumm 121) and 19 646 facts extracted from the summaries (INSIDE 10 768; BioLaySumm 8878) were analyzed by LLMs (Table 1). These facts when verified against the summaries, the average verification score (percentage of verified facts) ranged from 91.9% to 99.5% across LLMs for both datasets, suggesting accurate fact extraction. Average fact verification scores against the source title/abstracts were considerably lower for human-written PLS than INSIDE summaries for all LLMs (61.6% [95% confidence interval, CI, 60.1–63.1] vs 88.9% [95% CI, 88.0–89.8] for data excluding DeBERTa-v3-base) (Figure 2A and B). A higher hallucination rate was identified for human-written PLS than INSIDE summaries (40.6% [95% CI, 37.8–43.4] vs 9.6% [95% CI, 6.5–12.7] for data excluding DeBERTa-v3-base; Table S5). The verification scores decreased according to the severity of the perturbations for modified facts (*n* = 3228), with contradictory facts predominantly labeled as hallucination, suggesting accurate fact verification workflow. In contrast, all unmodified facts (*n* = 1076) were verifiable against the summaries (Figure 3). Overall, there was moderate agreement between all LLMs per the Fleiss kappa score of 0.40 [95% CI, 0.38–0.42], that is, 40.0% agreement.

### Faithfulness: fact verification by human experts

Out of 631 facts reviewed by any 3 human experts, 455 (77.0%) were checked by 2 reviewers and 77 (12.0%) were analyzed by 3 reviewers. Additionally, 99 (16.0%) facts that were reviewed by 1 judge only and 13.0% of facts labeled as “Unable to Judge” were excluded from analysis. There was higher inter-rater agreement among human experts for INSIDE (0.43 [95% CI, 0.34–0.52]) and BioLaySumm PLS (0.43 [95% CI, 0.330.53]) datasets compared to the INSIDE PLS (0.36 [95% CI, 0.280.44]). The maximum score for agreement between LLM panel and human experts panel (Cohen’s kappa 0.67 [95% CI, 0.61–0.73]) was observed when a panel of 4 LLMs agreed that the fact was faithful to the original title/abstract, suggesting that acceptance of facts by 4/5 LLMs maximizes agreement with the human panel (Figure S1A). The proportion of type-1 errors (claims supported by LLMs but deemed as hallucinations by experts) decreased with increase in the number of LLM used for fact verification (Figure S1B).

### Relevance

Overall, 100 documents (300 summaries) were assessed by the human experts and LLMs. There was an agreement on 40.0% of the rankings across all human experts. The linear weighted kappa scores were as follows: 0.36 [95% CI, 0.34–0.38] among LLMs, 0.11 [95% CI, 0.08–0.14] among the human experts, and 0.16 [95% CI, 0.13–0.19] between the LLM panel and human experts. These scores suggested that the panel of LLMs agreed more with the panel of human experts as compared to the individual human reviewers.

### Readability

**Figure 4.**
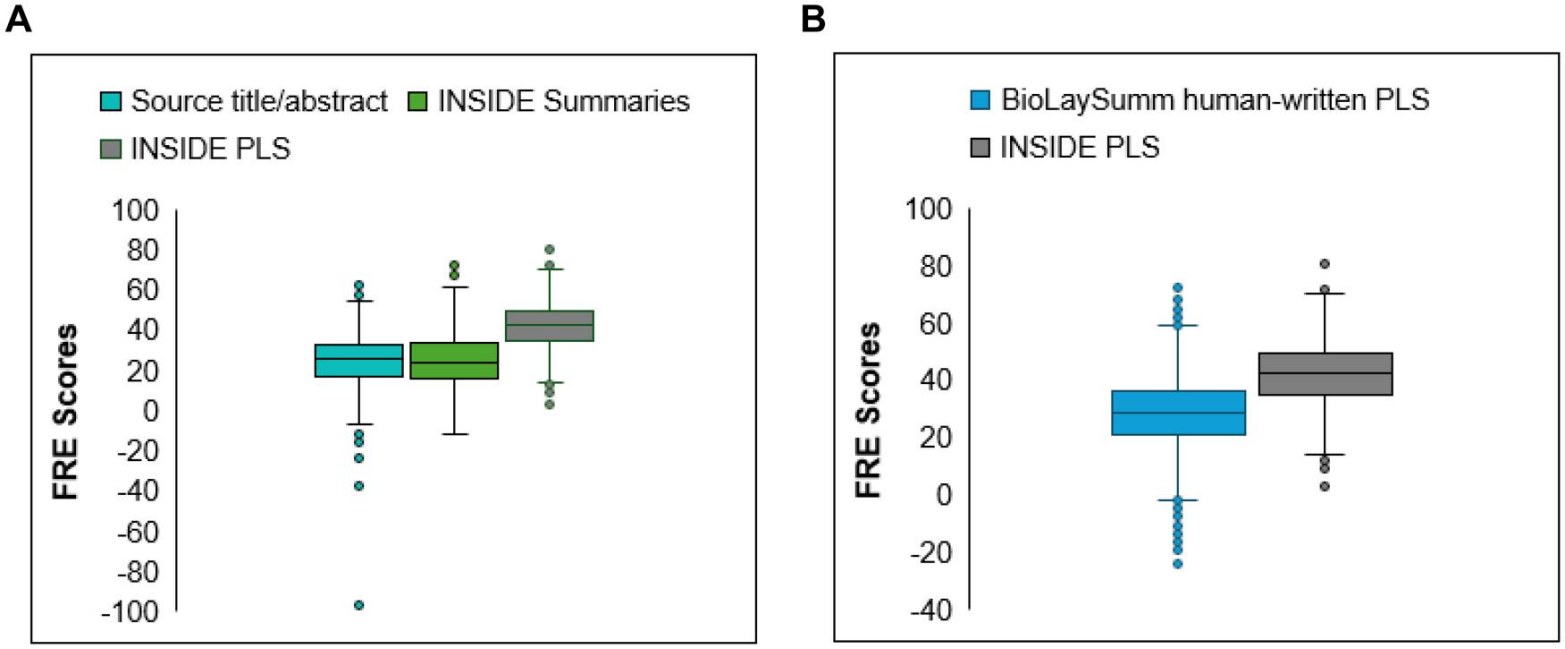
Comparison of readability scores of summaries. (A) FRE scores for LLM-generated summaries and PLS from the INSIDE dataset compared to original source/title abstracts. (B) FRE scores for human-written PLS from BioLaySumm compared to the LLM-generated PLS from INSIDE dataset. The negative FRE scores indicate that the text may be very difficult to read or possibly without an abstract. Abbreviations: FRE, Flesch-Kincaid Reading Ease; INSIDE, artificial INtelligence to Support Informed DEcision making; LLM, large language model; PLS, plain language summaries.

Publications from BioLaySumm (*n* = 24 759; all available PLS within the dataset), INSIDE PLS (*n* = 1000; large sample of INSIDE publications used for analysis), and the INSIDE scientific summaries (*n* = 1000) were considered for assessing readability. A comparison of random samples of 1000 summaries from the INSIDE dataset (source title/abstract vs INSIDE summaries vs INSIDE PLS) demonstrated higher FRE scores (representing readability) for INSIDE PLS (median 42.3 [interquartile range: IQR 35.27–49.41]) than source title/abstract (median 25.5 [IQR 17.01–33.24]). In comparison, the FRE scores for INSIDE summaries (median 24.1 [15.55–34.02]) were comparable to that of the source title/abstract (Figure 4A). Importantly, the readability scores for INSIDE PLS were higher compared to the human-written PLS from BioLaySumm (median 42.3 [IQR 35.27–49.41] vs 28.8 [IQR 21.02–36.18]; Figure 4B).

## Discussion

This study highlights a potential approach for evaluating hallucinations to demonstrate the quality and trustworthiness of LLM- vs human- generated summaries and PLS in clinical oncology. Additionally, the study focuses on best practices for assessment of clinical summaries using 3 key metrices—faithfulness, relevance, and readability. To our knowledge, this is the first study utilizing an automated approach to evaluate the quality of LLM-generated clinical summaries.

Given the objective of PLS is for enhanced patient understanding, the need for simplified language and translation of original clinical data to laymen terms in PLS may lead to potential misinterpretations by LLMs, resulting in inaccuracies or hallucinations. Our study results identified a higher rate of hallucinations in PLS authored by human experts compared to LLM-generated scientific summaries (40.6% vs 9.6%). While some of these differences might be due to distinct characteristics of the datasets (INSIDE vs BioLaySumm), the large effect size suggests extrinsic hallucinations in human-written PLS are potentially due to additional context and clarification added to PLS for better understanding by lay audience. This supplementary context is generally not included in LLM-generated scientific summaries, resulting in a much lower hallucination rate. A recent study comparing AI-generated (ChatGPT) and clinician-authored summaries based on 70 simulated patient electronic health records described that use of AI could result in more patient-friendly text and increase the chances of including more clinically relevant information.^19^ These findings support the potential of such AI summarization tools in generating accurate clinical summaries in a time-efficient manner for clinicians and patients and hence, should be optimized for further implementation in clinical practice.

For relevance assessment, human expert reviewers demonstrated a modest 40.0% agreement. This suggests the challenges and subjectivity in document ranking and relevance judgment even among human experts. However, a higher agreement level was noted between the panel of LLMs and human experts compared to individual human reviewers (0.16 [95% CI, 0.13–0.19] vs 0.11 [95% CI, 0.08–0.14]). While these findings should be interpreted with caution, given the modest human agreement, fine-tuning of LLMs using high-quality, oncology-specific, real-world training datasets would help to further improve their overall accuracy including relevance assessment.^20^ Our findings support future research focus on further enhancing relevance assessments in LLMs.

The readability analysis revealed higher FRE scores for LLM-generated PLS suggesting higher readability levels as compared to LLM-generated scientific summaries and original source documents, consistent with increased readability of PLS compared to clinical summaries. Importantly, higher readability scores for the LLM-generated PLS than those for human-written PLS suggests LLMs could generate PLS with better readability for lay audience when prompted appropriately (with specific instructions for desired output) as compared to human experts. Another study utilizing a specific AI approach comprising NLP and LLMs demonstrated significantly higher readability for LLM-generated PLS (*n* = 48) compared to human-written PLS (mean Flesch-Kincaid Grade level scores: 8.6 vs 12.3).^21^ Given the wider impact of relevant clinical literature for patients and nonspecialist readers including policy makers, patients, and caregivers, the low FRE scores for clinical summaries and high readability of LLM-generated PLS suggest the need for increasing the comprehensibility of such critical information and encourage the use of LLMs for PLS. Additionally, efficient automation of PLS generation with LLMs would reduce involvement of human experts leading to time- and cost-savings.

Based on the different metrics analyzed, our study proposes a holistic and interactive approach to represent the quality and trustworthiness of LLM-generated summaries. This visualization will present the original abstract as well as a note on the contested facts (ie, those facts not agreed upon by all LLMs) which are mapped to the original abstract allowing a user to either accept or reject that fact from the summary (Figure S2A). Additionally, a 3D visualization badge summarizing the scores for all 3 metrices will be presented for each summary (Figure S2B).

While LLMs offer powerful tools for generating clinical summaries, it is imperative to maintain ethical and clinical integrity.^9^^,^^22^ To ensure patient safety and wider application of LLMs in health care, robust detection and mitigation strategies for hallucinations are needed to avoid any potential errors due to missed information or misrepresentation of clinical data.^23–26^ To effectively monitor hallucinations, concrete measures such as involving humans in the loop for supervision, utilizing domain-specific metrics and methods for confidence estimation, training AI models with complex and diverse datasets to avoid bias, improving model architecture, prompt engineering strategies, and training and awareness of AI limitations will be helpful.^27^ Furthermore, establishing ethical guidelines and standards aligned with regulatory policies to evaluate performance benchmarks of such tools is crucial to manage hallucinations in clinical research.^9^^,^^23^

Common limitations of LLMs include lack of domain-based knowledge, privacy and data security concerns, data equity, transparency, data accuracy, and validation of AI tools.^9^^,^^20^^,^^28^ A fundamental challenge with addressing hallucinations is its diverse nature of errors which makes it difficult to standardize approaches and benchmarks for detection.^9^^,^^29^ The current analysis did not evaluate variety of prompt modifications to limit hallucinations. Additionally, current metrics often treat all hallucinations as equally problematic, without considering additional parameters to ascertain how harmful the hallucination is. Sometimes, extrinsic hallucinations that add context to the summary could be viewed as a necessity to improve relevance of the data presented in the summary.^30^ Sycophancy in LLMs which represents alignment with user inputs (containing potential inaccuracies) over accuracy of outputs was not assessed in this study.^31^ Low levels of agreement among human experts may have impacted the assessment of hallucinations. Therefore, developing a taxonomy of hallucinations should be considered to correctly estimate the “faithfulness” score. This study utilized the FRE score (a simple and widely used metric) to determine the readability of text, while there are other NLP-based metrics (eg, BERTScore, BLEU, ROUGE, METEOR) available.^32–35^ These formulas only provide estimates for readability of text and do not suggest how well the text is understood.^36^ Since external hallucinations typically occur in PLS, a method to automatically distinguish “ok” hallucinations from “not ok” ones will be helpful. Additionally, verification of a summary by multiple LLMs requires substantial cost and time, and therefore, an approach to determine whether a summary needs further assessment by LLM panel will be useful.

## Conclusion

This study demonstrates that LLMs could be used to automate the generation of scientific summaries and PLS with considerable levels of faithfulness, relevance, and readability from authentic data sources. Importantly, an LLM panel can serve as a useful tool to assess the faithfulness and transparency of summaries and identify certain facts that warrant further validation with the original source by readers. Additionally, LLM panel can facilitate estimation of relevance of a summary against the source title/abstract. When appropriately prompted, LLM-generated summaries tend to have better readability than those written by human experts. Independent verification by human-experts further substantiated the robustness of this multi-LLM assessment paradigm, demonstrating its utility in generating and validating accurate and contextually relevant summaries that inform clinical decision-making. Independent human expert review corroborated the robustness of the multi-LLM assessment paradigm, supporting its utility for generating and validating accurate, contextually relevant scientific and PLS. To our knowledge, this work is the first to implement a multi-LLM panel to systematically benchmark LLM-generated summaries of oncology literature, establishing a foundational methodology for mitigating hallucinations and enhancing confidence in AI-informed clinical knowledge dissemination. Given increasing information overload for clinicians and scientists, trustworthy summarization—paired with transparent verification—is a necessary foundation for responsible downstream use of LLM outputs in evidence consumption, and our framework operationalizes this need via faithfulness, relevance, and readability. Because our evaluation was performed in a controlled dataset environment, these findings should be interpreted as methodological evidence; prospective studies in real-world workflows are needed to quantify implementation impact.

## Supplementary Material

ooag078_Supplementary_Data

## Data Availability

The data underlying this article, such as LLM prompts, are available in Supplementary Material S1 accompanying this article. Additional details will be shared on reasonable request to the corresponding author.
