# Supplementary material for: Enhancing the quality and trustworthiness of large language model-generated summaries of clinical oncology literature
Source: JAMIA Open. 2026 Jun 16;9(3):ooag078. doi: 10.1093/jamiaopen/ooag078 (PMC13275010; doi:10.1093/jamiaopen/ooag078)
Supplement: ooag078_Supplementary_Data [file ooag078_supplementary_data.zip › Supplementary File_Clean.docx]

**Supplementary File:**

## **Supplementary Methods:**

**LLMs**

A Natural Language Inference (NLI) model based on DeBERTa-v3-base was used to assess if a smaller cost-effective model could provide similar levels of agreement as other LLMs or human experts.^1^ This was used for comparison of fact verification.

**Human Experts**

The panel of human reviewers comprised clinical experts across multiple disciplines spanning surgical practice, epidemiologic research, and oncology—for a multifaceted perspective on the evaluation of LLM-generated content for faithfulness and relevance.

A workshop was performed with the human experts where instructions for the task were presented and subsequent questions from the human expert reviewers on the scope of their assessments were answered. The reviewers then performed their assessment independently.

**Inter-rater Reliability**

This was used to quantify the degree of agreement among individual LLMs and human experts, as well as between the LLM panel and human experts for evaluating faithfulness and relevance. While Cohen’s kappa statistics (κ) measured agreement between two raters,^2^ Fleiss kappa is an adaptation of Cohen’s kappa which was used for three or more raters.^3^ A linear weighted kappa score which penalizes higher discrepancies between rankings and provides more information about inter-rater agreement was additionally used for analyzing relevance of summaries.^2^

**Readability**

The Flesch-Kincaid Reading Ease formula reflects the reader’s ability to understand a given body of text based on the average number of words and syllables and generates a corresponding score on a scale of 0–100 based on the readability of the text. The higher the score, the more is the ease of reading.^4,5^

**LLM frameworks, software, parameters and prompt strategies**

We have used opensource framework langchain (version 0.3.37) for all LLM experiments.^6^ The underlying models were used with default parameters by specific providers (OpenAI, Bedrock, and Vertex AI). Exact model ids passed to langchain are listed in the table below. The same prompts (as described in Table S2) have been used with all providers. To improve the output of the models, we carefully designed the prompts to include clear content guidelines, delimitation, and precise output descriptions.

## **Table S1: LLM Frameworks Used for Analyses**

| **Summarization models:** | |
| --- | --- |
| **Model** | **Model ID** |
| GPT-4o | gpt-4o |
| GPT-4o-mini | gpt-4o-mini |
| Gemini-1.5-Flash-002 | gemini-1.5-flash-002 |
| Gemini-1.5-Pro-002 | gemini-1.5-pro-002 |
| Claude-3-Haiku | anthropic.claude-3-haiku-20240307-v1:0 |
| Claude-3.5-Sonnet | us.anthropic.claude-3-5-sonnet-20240620-v1:0 |
| Llama-3.2-1B | us.meta.llama3-2-1b-instruct-v1:0 |
| Llama-3-70B | us.meta.llama3-1-70b-instruct-v1:0 |
| Mistral-8x7B | mistral.mixtral-8x7b-instruct-v0:1 |
| Mistral-Large | mistral.mistral-large-2402-v1:0 |
| **Fact extraction models:** | |
| **Model** | **Model ID** |
| GPT-4o | gpt-4o |
| Gemini-1.5-Pro-002 | gemini-1.5-pro-002 |
| Claude-3.5-Sonnet | us.anthropic.claude-3-5-sonnet-20240620-v1:0 |
| Llama-3-70B | us.meta.llama3-1-70b-instruct-v1:0 |
| Mistral-8x7B | mistral.mixtral-8x7b-instruct-v0:1 |
| **Fact verification models:** | |
| **Model** | **Model ID** |
| GPT-4o | gpt-4o |
| Gemini-1.5-Pro-002 | gemini-1.5-pro-002 |
| Claude-3.5-Sonnet | us.anthropic.claude-3-5-sonnet-20240620-v1:0 |
| Llama-3-70B | us.meta.llama3-1-70b-instruct-v1:0 |
| Mistral-8x7B | mistral.mixtral-8x7b-instruct-v0:1 |
| DeBERTa-3-base | MoritzLaurer/DeBERTa-v3-base-mnli-fever-docnli-ling-2c |

## **Table S2: Prompts Used for Different Analyses**

| **Summarization:** |
| --- |
| **Prompt Summary (INSIDE Scientific)**  Below is a document representing the title and abstract of a scientific publication in health sciences.  The end goal is to summarize it for readers with extensive medical background.  You can use well established abbreviations without writing out the long form.  Analyze the document text and make sure the below elements are in the summary if they are mentioned in the document.  - The central theme or argument  - Key supporting ideas  - Important facts or evidence  - Clinical statements about treatments, adverse events, outcomes  - Purpose or perspective of the study  - Significant implications or conclusions  The summary should be approximately 750 characters, or half the size of the abstract, whatever is smallest.  Answer only in JSON in the following format:  {format_instructions}  Document:  {document} |
| **Prompt Summary (INSIDE PLS)**  Below is a document representing the title and abstract of a scientific publication in health sciences.  The end goal is to summarize it for readers with no scientific background.  Aim to create a summary with a The Flesch–Kincaid reading grade level of above 50 (i.e. understandable by 10th to 12th grade students).  Analyze the document text and make sure the below elements are in the summary if they are mentioned in the document.  - The central theme or argument  - Key supporting ideas  - Important facts or evidence  - Clinical statements about treatments, adverse events, outcomes  - Purpose or perspective of the study  - Significant implications or conclusions  The summary should be approximately 750 characters, or half the size of the abstract, whatever is smallest.  Answer only in JSON in the following format:  {format_instructions}  Document:  {document} |
| **Fact extraction:** |
| **Prompt fact extraction**  Below is a summary of a document. Please extract ALL the atomic facts from the document.  Guidelines:  - An atomic fact is a single individually verifiable statement.  - Each sentence may contain multiple atomic facts.  - You are allowed to split up sentences into multiple facts.  - Ensure that every fact is complete and verifiable (e.g. no coreferences in facts).  Summary:  {summary}  {format_instructions}  IMPORTANT: only answer in JSON |
| **Fact modification (subtle, moderate, and contradictory):** |
| **Prompt fact modification (subtle)**  I want you act as a hallucination text generator.  Given an atomic claim, you hallucinate another claim.  You SHOULD write the hallucinated claim using the following method:  You are generating a claim similar to the original, but it is different from the original atomic claim in a subtle way.  For instance, the confidence expressed in the claim is slightly different, causing a probable to become possible or highly probable, or a p-value of 0.1 to become 0.5 or 0.03, etc.  Claim:  {fact}  You output only JSON in the following format:  {format_instructions} |
| **Prompt fact modification (medium)**  I want you to act as a hallucination text generator.  Given an atomic claim, generate another claim that is similar in structure but differs significantly in meaning.  Follow these guidelines:  * The hallucinated claim should retain all the noun phrases from the original claim.  * Use verbs that are similar to those in the original claim.  * Ensure the new claim is neither contradictory to the original nor a subtle variation.  * Avoid making changes that simply alter the level of confidence (e.g., probable to possible) or p-values (e.g., 0.1 to 0.5).  You output only JSON in the following format:  {format_instructions}  Claim:  {facts} |
| **Prompt fact modification (contradictory)**  I want you act as a hallucination text generator.  Given an atomic claim, you hallucinate another claim.  You SHOULD write the hallucinated claim using the following method:  You are generating a claim similar to the original, but it is a contradiction to the atomic claim.  You output only JSON in the following format:  {format_instructions}  Claim:  {facts} |
| **Fact verification:** |
| **Prompt fact verification (batch)**  Below is a document and a list of claims.  Check for each claim whether it is supported by the document.  Respond with the claim number and a value true or false.  Document:  {document}  Claims:  {claims}  Give your answer in the following format:  {format_instructions}  IMPORTANT:  - include quotes in your reasoning!  - only answer with the JSON format. No surrounding text! |
| **Relevance Analysis** |
| **Prompt Summary relevance modification (severe)**  Below is a document representing the title and abstract of a scientific publication in health sciences.  The end goal is to summarize it for readers with a casual interest in the topic.  You can use well established abbreviations without writing out the long form.  Analyze the document text and ensure the below elements are in the summary if they are mentioned in the document:  • Any tangential or anecdotal details, even if unrelated to the central argument  • Minor observations or background context from the abstract  Ensure you exclude major conclusions, central themes, or key supporting ideas entirely.  The summary should be approximately 750 characters, or half the size of the abstract, whatever is smallest.  Answer only in JSON in the following format:  {format_instructions}  Document:  {document} |
| **Prompt summary relevance modification (moderate)**  Below is a document representing the title and abstract of a scientific publication in health sciences.  The end goal is to summarize it for readers with general scientific background.  You can use well established abbreviations without writing out the long form.  Analyze the document text and ensure the below elements are in the summary if they are mentioned in the document:  • The central theme or argument  • Clinical statements about treatments, adverse events, outcomes  • Purpose or perspective of the study  Do not include detailed supporting ideas or evidence used in the study. Also exclude significant implications or conclusions.  The summary should be approximately 750 characters, or half the size of the abstract, whatever is smallest.  Answer only in JSON in the following format:  {format_instructions}  Document:  {document} |

## **Table S3: Labels Used for Fact verification by Human Experts**

| **Labels** | **Definition and Examples** |
| --- | --- |
| **Fact ok** | The fact is entirely accurate and aligns perfectly with the corresponding information in the abstract.  **E.g.** If the abstract reports “35% of patients showed improvement”, and the fact states “35% of patients improved” |
| **Fact partly ok** | The fact is partially accurate with minor discrepancies or missing context, but is not entirely misleading  **E.g.** If the abstract reports “35% of patients aged 25–40 improved”, but the fact states “35% of patients improved” |
| **Unable to judge** | There is insufficient information to confirm or deny the accuracy of the fact.  **E.g.** If the fact states ‘The study included a control group”, but the abstract does not mention a control group. |
| **Hallucination** | The fact is fabricated or contains information that is not present anywhere in the abstract.  **E.g.** If the fact mentions “50% success rate”, but the abstract has no discussion or data about success rates. |
| **Nonsense** | The fact is incoherent, irrelevant, or illogical.  **E.g.** If the fact states “This is an example fact”. |

## **Table S4: Prompts Used for Creating Three Levels of Relevance**

| **Level of relevance** | **Type of prompt used** |
| --- | --- |
| Original (as relevant as possible) | The prompt states to include all important elements in the summary |
| Moderate irrelevance | The prompt states to exclude detailed supporting ideas or evidence used in the study, and also significant implications or conclusions |
| Severe irrelevance | The prompt states to exclude major conclusions, central themes, or key supporting ideas entirely, and include tangential or anecdotal details, even if unrelated to the central argument, and minor observations or background context |

## **Table S5: Hallucination Rate per Document in INSIDE and BioLaySumm Datasets**

| doc_id | percentage_fact_ok | dataset |
| --- | --- | --- |
| pub.1002671071 | 0.9 | INSIDE |
| pub.1003695905 | 0.99 | INSIDE |
| pub.1009170398 | 0.83 | INSIDE |
| pub.1009295271 | 0.89 | INSIDE |
| pub.1014532016 | 0.9 | INSIDE |
| pub.1016206684 | 0.98 | INSIDE |
| pub.1018681126 | 0.88 | INSIDE |
| pub.1019090430 | 1 | INSIDE |
| pub.1019705710 | 0.8 | INSIDE |
| pub.1023328349 | 0.88 | INSIDE |
| pub.1025160751 | 0.95 | INSIDE |
| pub.1035344380 | 0.99 | INSIDE |
| pub.1035386940 | 0.92 | INSIDE |
| pub.1038779893 | 0.93 | INSIDE |
| pub.1042057124 | 0.94 | INSIDE |
| pub.1045710692 | 0.99 | INSIDE |
| pub.1049999774 | 0.98 | INSIDE |
| pub.1064076988 | 0.98 | INSIDE |
| pub.1085096696 | 0.94 | INSIDE |
| pub.1085572993 | 0.9 | INSIDE |
| pub.1085714330 | 0.977777778 | INSIDE |
| pub.1090741729 | 0.31 | INSIDE |
| pub.1091305961 | 0.99 | INSIDE |
| pub.1093070170 | 0.95 | INSIDE |
| pub.1099686765 | 0.94 | INSIDE |
| pub.1104512661 | 0.87 | INSIDE |
| pub.1105738329 | 0.97 | INSIDE |
| pub.1107586744 | 0.9 | INSIDE |
| pub.1107906809 | 0.95 | INSIDE |
| pub.1110370514 | 0.87 | INSIDE |
| pub.1110581882 | 0.69 | INSIDE |
| pub.1117622452 | 0.9 | INSIDE |
| pub.1120409735 | 0.95 | INSIDE |
| pub.1121256830 | 1 | INSIDE |
| pub.1121390260 | 0.98 | INSIDE |
| pub.1121552706 | 0.99 | INSIDE |
| pub.1122486182 | 0.97 | INSIDE |
| pub.1123670599 | 0.95 | INSIDE |
| pub.1124346468 | 0.95 | INSIDE |
| pub.1124948832 | 0.77 | INSIDE |
| pub.1126153308 | 0.85 | INSIDE |
| pub.1127167893 | 0.91 | INSIDE |
| pub.1127453098 | 0.91 | INSIDE |
| pub.1127727052 | 0.91 | INSIDE |
| pub.1129830564 | 0.91 | INSIDE |
| pub.1130128322 | 0.83 | INSIDE |
| pub.1130146683 | 0.88 | INSIDE |
| pub.1131002285 | 0.91 | INSIDE |
| pub.1131399791 | 0.97 | INSIDE |
| pub.1131624717 | 0.81 | INSIDE |
| pub.1132048509 | 0.85 | INSIDE |
| pub.1132882194 | 0.92 | INSIDE |
| pub.1132940447 | 0.95 | INSIDE |
| pub.1133120204 | 0.93 | INSIDE |
| pub.1134139280 | 0.91 | INSIDE |
| pub.1134145009 | 0.84 | INSIDE |
| pub.1134475789 | 0.99 | INSIDE |
| pub.1135005169 | 0.84 | INSIDE |
| pub.1135100603 | 0.93 | INSIDE |
| pub.1135474716 | 1 | INSIDE |
| pub.1135587036 | 0.88 | INSIDE |
| pub.1136474624 | 0.87 | INSIDE |
| pub.1136686202 | 0.87 | INSIDE |
| pub.1136702599 | 0.86 | INSIDE |
| pub.1137538277 | 0.88 | INSIDE |
| pub.1138023259 | 0.98 | INSIDE |
| pub.1138206960 | 0.285714286 | INSIDE |
| pub.1138298045 | 0.97 | INSIDE |
| pub.1138730098 | 0.78 | INSIDE |
| pub.1138891413 | 0.92 | INSIDE |
| pub.1138972508 | 0.93 | INSIDE |
| pub.1139077509 | 0.98 | INSIDE |
| pub.1139414985 | 0.92 | INSIDE |
| pub.1139648769 | 0.93 | INSIDE |
| pub.1141107478 | 0.84 | INSIDE |
| pub.1141211391 | 0.79 | INSIDE |
| pub.1142338040 | 0.96 | INSIDE |
| pub.1142345269 | 0.95 | INSIDE |
| pub.1142520430 | 0.82 | INSIDE |
| pub.1142605911 | 0.91 | INSIDE |
| pub.1143509727 | 0.88 | INSIDE |
| pub.1143955736 | 0.86 | INSIDE |
| pub.1145042326 | 0.81 | INSIDE |
| pub.1145278503 | 1 | INSIDE |
| pub.1145509687 | 0.92 | INSIDE |
| pub.1145832459 | 0.88 | INSIDE |
| pub.1147189021 | 0.92 | INSIDE |
| pub.1147254594 | 0.89 | INSIDE |
| pub.1147356554 | 0.99 | INSIDE |
| pub.1147568625 | 0.9 | INSIDE |
| pub.1147860116 | 0.99 | INSIDE |
| pub.1147988809 | 0.96 | INSIDE |
| pub.1148000034 | 0.94 | INSIDE |
| pub.1148263418 | 0.97 | INSIDE |
| pub.1148314658 | 0.97 | INSIDE |
| pub.1148363737 | 0.966666667 | INSIDE |
| pub.1148715493 | 0.92 | INSIDE |
| pub.1148883476 | 0.86 | INSIDE |
| pub.1150156951 | 0.88 | INSIDE |
| pub.1151427929 | 0.93 | INSIDE |
| pub.1151935483 | 0.9 | INSIDE |
| pub.1151993869 | 0.96 | INSIDE |
| pub.1152238161 | 0.89 | INSIDE |
| pub.1152311989 | 0.92 | INSIDE |
| pub.1152736018 | 1 | INSIDE |
| journal.pbio.1000121 | 0.494117647 | BioLaySumm |
| journal.pbio.1000275 | 0.5 | BioLaySumm |
| journal.pbio.1000319 | 0.695652174 | BioLaySumm |
| journal.pbio.1000563 | 0.642857143 | BioLaySumm |
| journal.pbio.1001363 | 0.511363636 | BioLaySumm |
| journal.pbio.1001461 | 0.565217391 | BioLaySumm |
| journal.pbio.2000487 | 0.87804878 | BioLaySumm |
| journal.pbio.2000784 | 0.408450704 | BioLaySumm |
| journal.pbio.2002930 | 0.587301587 | BioLaySumm |
| journal.pbio.2005907 | 0.819672131 | BioLaySumm |
| journal.pbio.2006134 | 0.402985075 | BioLaySumm |
| journal.pbio.3000051 | 0.333333333 | BioLaySumm |
| journal.pbio.3000228 | 0.698630137 | BioLaySumm |
| journal.pcbi.0030161 | 0.393939394 | BioLaySumm |
| journal.pcbi.1000138 | 0.3 | BioLaySumm |
| journal.pcbi.1000712 | 0.566666667 | BioLaySumm |
| journal.pcbi.1001114 | 0.5 | BioLaySumm |
| journal.pcbi.1001123 | 0.525 | BioLaySumm |
| journal.pcbi.1002143 | 0.884057971 | BioLaySumm |
| journal.pcbi.1002206 | 0.592105263 | BioLaySumm |
| journal.pcbi.1002227 | 0.756756757 | BioLaySumm |
| journal.pcbi.1002240 | 0.650793651 | BioLaySumm |
| journal.pcbi.1002875 | 0.666666667 | BioLaySumm |
| journal.pcbi.1002975 | 0.568965517 | BioLaySumm |
| journal.pcbi.1003008 | 0.580246914 | BioLaySumm |
| journal.pcbi.1003054 | 0.5 | BioLaySumm |
| journal.pcbi.1003086 | 0.461538462 | BioLaySumm |
| journal.pcbi.1003100 | 0.591397849 | BioLaySumm |
| journal.pcbi.1003120 | 0.15 | BioLaySumm |
| journal.pcbi.1003358 | 0.728571429 | BioLaySumm |
| journal.pcbi.1003409 | 0.432835821 | BioLaySumm |
| journal.pcbi.1003545 | 0.605633803 | BioLaySumm |
| journal.pcbi.1003665 | 0.71875 | BioLaySumm |
| journal.pcbi.1003895 | 0.735632184 | BioLaySumm |
| journal.pcbi.1004021 | 0.892857143 | BioLaySumm |
| journal.pcbi.1004024 | 0.5625 | BioLaySumm |
| journal.pcbi.1004042 | 0.629032258 | BioLaySumm |
| journal.pcbi.1004050 | 0.791044776 | BioLaySumm |
| journal.pcbi.1004115 | 0.61971831 | BioLaySumm |
| journal.pcbi.1004199 | 0.425925926 | BioLaySumm |
| journal.pcbi.1004247 | 0.472222222 | BioLaySumm |
| journal.pcbi.1004269 | 0.320512821 | BioLaySumm |
| journal.pcbi.1004272 | 0.454545455 | BioLaySumm |
| journal.pcbi.1004293 | 0.694915254 | BioLaySumm |
| journal.pcbi.1004426 | 0.484848485 | BioLaySumm |
| journal.pcbi.1004518 | 0.303571429 | BioLaySumm |
| journal.pcbi.1004578 | 0.655737705 | BioLaySumm |
| journal.pcbi.1004595 | 0.533333333 | BioLaySumm |
| journal.pcbi.1004827 | 0.637681159 | BioLaySumm |
| journal.pcbi.1005049 | 0.867924528 | BioLaySumm |
| journal.pcbi.1005125 | 0.770491803 | BioLaySumm |
| journal.pcbi.1005509 | 0.5625 | BioLaySumm |
| journal.pcbi.1005793 | 0.634615385 | BioLaySumm |
| journal.pcbi.1005890 | 0.723809524 | BioLaySumm |
| journal.pcbi.1005985 | 0.476744186 | BioLaySumm |
| journal.pcbi.1006657 | 0.539473684 | BioLaySumm |
| journal.pcbi.1006730 | 0.416666667 | BioLaySumm |
| journal.pcbi.1006981 | 0.609375 | BioLaySumm |
| journal.pcbi.1007239 | 0.739130435 | BioLaySumm |
| journal.pcbi.1007344 | 0.807692308 | BioLaySumm |
| journal.pgen.1000080 | 0.521126761 | BioLaySumm |
| journal.pgen.1000129 | 0.746031746 | BioLaySumm |
| journal.pgen.1000293 | 0.644067797 | BioLaySumm |
| journal.pgen.1000850 | 0.8 | BioLaySumm |
| journal.pgen.1001351 | 0.616438356 | BioLaySumm |
| journal.pgen.1002042 | 0.631067961 | BioLaySumm |
| journal.pgen.1002182 | 0.54 | BioLaySumm |
| journal.pgen.1002218 | 0.505882353 | BioLaySumm |
| journal.pgen.1002360 | 0.652631579 | BioLaySumm |
| journal.pgen.1002368 | 0.852459016 | BioLaySumm |
| journal.pgen.1002538 | 0.59375 | BioLaySumm |
| journal.pgen.1002689 | 0.328767123 | BioLaySumm |
| journal.pgen.1002735 | 0.623188406 | BioLaySumm |
| journal.pgen.1003876 | 0.846153846 | BioLaySumm |
| journal.pgen.1003997 | 0.37962963 | BioLaySumm |
| journal.pgen.1004135 | 0.592105263 | BioLaySumm |
| journal.pgen.1004173 | 0.602739726 | BioLaySumm |
| journal.pgen.1004453 | 0.44 | BioLaySumm |
| journal.pgen.1004475 | 0.794871795 | BioLaySumm |
| journal.pgen.1004520 | 0.763888889 | BioLaySumm |
| journal.pgen.1004809 | 0.397435897 | BioLaySumm |
| journal.pgen.1005228 | 0.656716418 | BioLaySumm |
| journal.pgen.1005334 | 0.554347826 | BioLaySumm |
| journal.pgen.1005633 | 0.703296703 | BioLaySumm |
| journal.pgen.1005755 | 0.545454545 | BioLaySumm |
| journal.pgen.1005778 | 0.768115942 | BioLaySumm |
| journal.pgen.1005955 | 0.447368421 | BioLaySumm |
| journal.pgen.1006208 | 0.473684211 | BioLaySumm |
| journal.pgen.1006244 | 0.809917355 | BioLaySumm |
| journal.pgen.1006248 | 0.690140845 | BioLaySumm |
| journal.pgen.1006267 | 0.35483871 | BioLaySumm |
| journal.pgen.1006279 | 0.639344262 | BioLaySumm |
| journal.pgen.1006306 | 0.461538462 | BioLaySumm |
| journal.pgen.1006589 | 0.780487805 | BioLaySumm |
| journal.pgen.1006691 | 0.722891566 | BioLaySumm |
| journal.pgen.1006748 | 0.254237288 | BioLaySumm |
| journal.pgen.1006973 | 0.858974359 | BioLaySumm |
| journal.pgen.1006990 | 0.37254902 | BioLaySumm |
| journal.pgen.1007109 | 0.617283951 | BioLaySumm |
| journal.pgen.1007352 | 0.743589744 | BioLaySumm |
| journal.pgen.1007355 | 0.470588235 | BioLaySumm |
| journal.pgen.1007571 | 0.549450549 | BioLaySumm |
| journal.pgen.1007589 | 0.593406593 | BioLaySumm |
| journal.pgen.1007611 | 0.482758621 | BioLaySumm |
| journal.pgen.1007687 | 0.519480519 | BioLaySumm |
| journal.pgen.1007802 | 0.453488372 | BioLaySumm |
| journal.pgen.1007849 | 0.525423729 | BioLaySumm |
| journal.pgen.1008227 | 0.333333333 | BioLaySumm |
| journal.pgen.1008344 | 0.862068966 | BioLaySumm |
| journal.ppat.0040035 | 0.710843373 | BioLaySumm |
| journal.ppat.1000389 | 0.510638298 | BioLaySumm |
| journal.ppat.1000578 | 0.557142857 | BioLaySumm |
| journal.ppat.1000742 | 0.72972973 | BioLaySumm |
| journal.ppat.1001076 | 0.616666667 | BioLaySumm |
| journal.ppat.1002014 | 0.682926829 | BioLaySumm |
| journal.ppat.1003717 | 0.32183908 | BioLaySumm |
| journal.ppat.1006440 | 0.75 | BioLaySumm |
| journal.ppat.1006649 | 0.589285714 | BioLaySumm |
| journal.ppat.1006653 | 0.682926829 | BioLaySumm |
| journal.ppat.1006844 | 0.878378378 | BioLaySumm |
| journal.ppat.1007276 | 0.685714286 | BioLaySumm |

## **Fig. S1: Comparison of LLM Agreement with Human Experts.** Panel A shows agreement between LLM panel and human experts panel with maximum score achieved when 4/5 LLMs accept faithfulness of a fact. Panel B shows minimized type 1 error with the use of 5 LLMs. LLMs denote large language models.


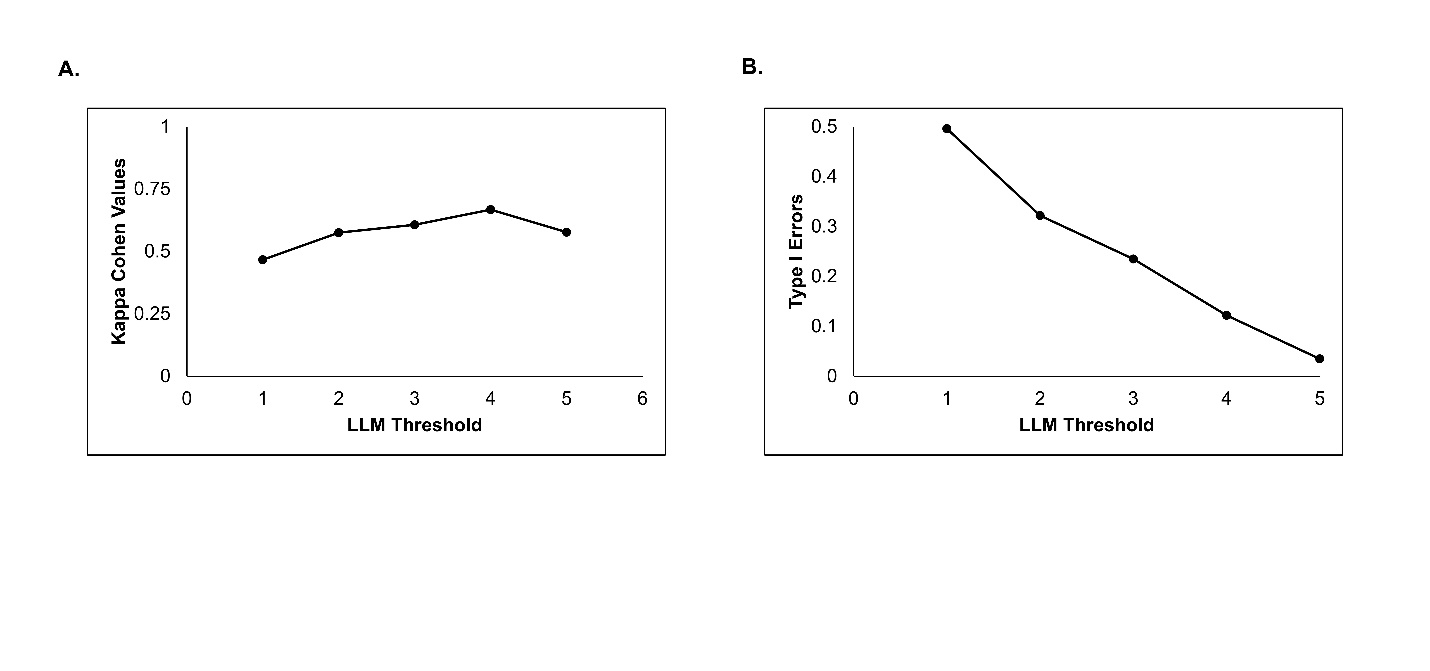


## **Fig. S2: Interactive Visualization of Summary Based on Quality Metrics.** Panel A shows original abstract summary indicating facts not agreed upon by all LLMs. Panel B shows scores for different quality metrics (readability, faithfulness, and relevance). LLMs denote large language models.

A.


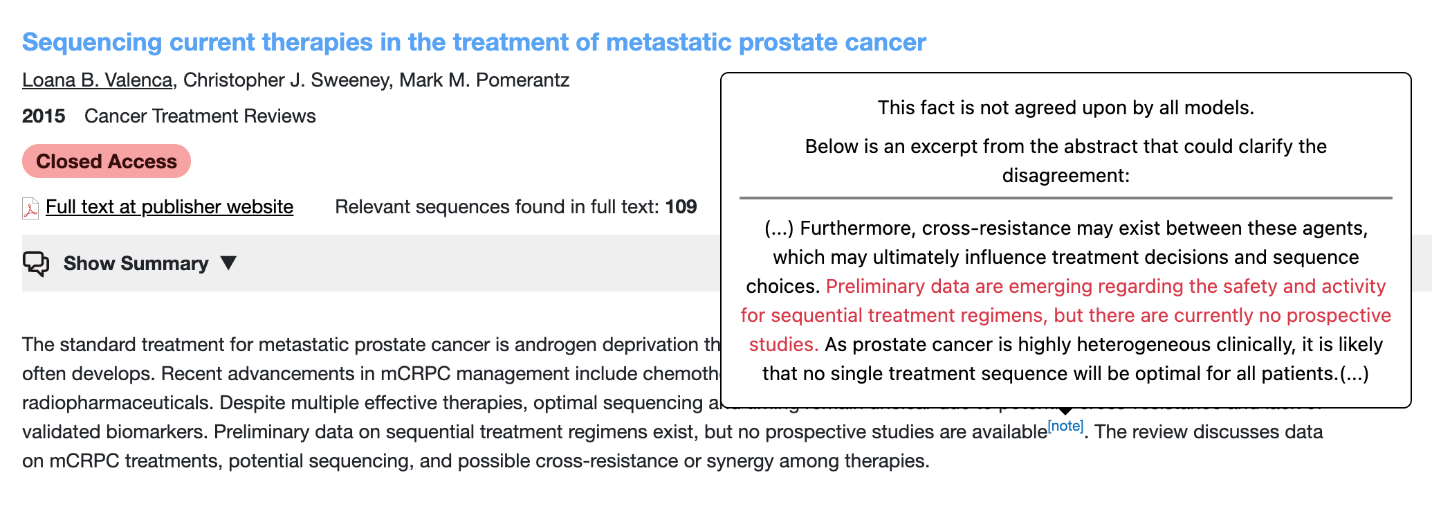


B.


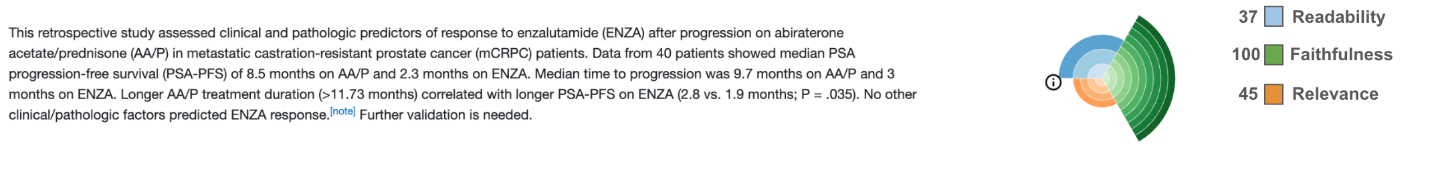


**References:**

1. He P, Gao J, Chen W. Debertav3: Improving deberta using electra-style pre-training with gradient-disentangled embedding sharing. November 18, 2021 (<https://doi.org/10.48550/arXiv.2111.09543>). Preprint.
2. Li M, Gao Q, Yu T. Kappa statistic considerations in evaluating inter-rater reliability between two raters: which, when and context matters. *BMC Cancer.* 2023;23:799.
3. McHugh ML. Interrater reliability: the kappa statistic. *Biochem Med (Zagreb)*. 2012;22:276-282.
4. Kincaid P, Fishburne RP, Rogers RL, Chissom BS. Derivation of New Readability Formulas (Automated Readability Index, Fog Count and Flesch Reading Ease Formula) for Navy Enlisted Personnel. 1975 (<https://stars.library.ucf.edu/istlibrary>).
5. Jindal P, MacDermid JC. Assessing reading levels of health information: uses and limitations of flesch formula. Educ Health (Abingdon) 2017;30:84-88.
6. Chase, Harrison. LangChain. version 0.3.37, 2022, <https://github.com/langchain-ai/langchain>
